# Supplementary material for: Are carbon emissions trading and green financial instruments synergistic? -Comprehensive quantitative research based on content analysis
Source: PLoS One. 2024 Mar 7;19(3):e0298601. doi: 10.1371/journal.pone.0298601 (PMC10919601; doi:10.1371/journal.pone.0298601)
Supplement: S1 Data — (ZIP) [file pone.0298601.s001.zip › Data/地级市Z_F工作报告.zip!/╣π╢1⁄2/╣π╓▌╩╨/└┤╫╘╣1⁄2╓┌║┼í╛┬φ┐╦╩2╛▌═°í┐_2012.docx]

各位代表：

　　我代表广州市人民政府，向大会作工作报告，请予审议，并请政协委员和其他列席人员提出意见。

　　一、过去五年和2011年工作回顾

　　本届政府任期的五年，是我市科学发展取得重大成就的五年。在党中央、国务院和省委、省政府及市委的正确领导下，在市人大和市政协的监督支持下，市政府依法行政，积极履行职责，和全市人民一道，以邓小平理论、“三个代表”重要思想为指导，深入贯彻落实科学发展观，大力实施《珠江三角洲地区改革发展规划纲要（2008－2020年）》（以下简称《规划纲要》），积极应对国际金融危机和严重自然灾害等严峻挑战，全力调结构、促转变、谋发展、惠民生，改革开放和现代化建设取得重大成就，市十三届人大各次会议确定的主要目标任务圆满完成。

　　五年来，经济发展实现新跨越，综合实力迈上新台阶。

　　经济实力大幅提升。2011年，预计实现地区生产总值12380亿元，同比增长11％，是2006年的1.8倍。预计源于广州地区的财政一般预算收入3965亿元，同比增长18.4％，其中，全市地方财政一般预算收入960亿元，同比增长18.1％，分别是2006年的2.3倍和2.4倍。社会消费品零售总额5220亿元，同比增长16.5％，是2006年的2.4倍。预计全市金融机构存贷款余额分别达25800亿元和17800亿元，是2006年的1.93倍和2.05倍。

　　结构调整步伐加快。坚持产业高端发展，全力建设现代产业体系，预计三次产业结构由2006年的2.1：40.2：57.7 调整为2011年的1.6：36.9：61.5。金融保险、商贸会展、现代物流、文化旅游等现代服务业蓬勃发展，服务业对经济增长的贡献率达60.5％，比2006年增加3.7个百分点，以服务经济为主体的产业结构特征更加凸显。汽车、造船、重大装备等先进制造业进一步发展壮大，预计2011年规模以上工业总产值15750亿元，同比增长12.5％，是2006年的2倍。高新技术产业加快发展，预计规模以上工业高新技术产品产值6311亿元，占规模以上工业总产值比重达40％，比2006年提高10个百分点。都市型现代农业稳步发展，预计农业总产值349亿元，同比增长3％。市属国有企业结构调整和转型升级成效显著，经济效益提高。民营企业健康发展，预计2011年民营经济实现增加值4750亿元，占全市地区生产总值比重达38.4％，同比增长11.5％，高于全市经济增速0.5个百分点。“退二进三”和“双转移”成效明显，关停、转移、淘汰各类企业6119家。五年累计安排重大产业项目89个，投资总额5005亿元，经济发展后劲进一步增强。“十一五”节能减排任务超额完成，预计2011年全市单位地区生产总值能耗同比下降4％以上，各项减排任务均可超额完成年度计划。

　　科技创新取得突破。不断加大研发投入，预计2011年全社会研发投入占地区生产总值比重达2.25％，比2006年提高0.55个百分点。合作建设11个国家级、23个省级国际科技合作基地，建成工业技术研究院等12个产学研创新平台，形成了新型显示、节能环保、移动互联网等10个百亿级创新集群。完善人才政策，发挥留交会平台作用，引进13个国际领先科研创新团队和25名领军人才，拥有各类博士后工作站55个，比2006年增长111.5％。知识产权创造能力进一步提升，2011年全市发明专利申请量和发明专利授权量分别达8172件和3146件，同比增长25.7％和58.2％，是2006年的3.0倍和4.5倍。全市信息化发展指数达到0.946，互联网普及率超过70％，政府管理和公共服务基本实现网络化，电子商务交易额居全国城市前列。2011年荣获“中国十大创新型城市”称号。

　　开放型经济发展水平提高。对外贸易平稳发展，预计2011年实现进出口总额超过1150亿美元，同比增长11％，是2006年的1.8倍；其中出口561亿美元，同比增长16％，是2006年的1.73倍。利用外资规模不断扩大，五年累计吸收合同外资284.3亿美元，实际利用外资189.2亿美元，其中预计2011年吸收合同外资67.2亿美元，同比增长35％；实际利用外资42.6亿美元，同比增长7％。世界500强企业有224家落户我市。举办“新广州·新商机”系列招商推介会，共签约400多个项目。积极实施“走出去”战略，我市企业五年累计对外投资23.66亿美元，境外投资领域进一步拓宽。穗港澳台合作不断深化，建立了穗港澳合作协调长效机制。南沙开发区成为全省实施CEPA先行先试综合示范区。

　　经济增长极带动作用增强。广州开发区主要经济效益指标连续多年位居全国开发区前列，增城工业园升级为国家级经济技术开发区，南沙新区开发建设上升为国家发展战略。预计2011年三大国家级开发区共实现规模以上工业总产值6855亿元，占全市43.5％，成为推动全市经济转型升级的重要平台。中新广州知识城、科学城、天河智慧城、国际生物岛等自主创新核心载体建设取得突破，现代服务业功能区、先进制造业基地、国家高新技术产业基地、空港经济区等产业功能区建设步伐加快，经济增长的引擎作用进一步强化。

　　五年来，举全市之力办成了一系列大事，城市影响力显著增强。

　　“两个亚运”圆满精彩。以“迎接亚运会，创造新生活”为主题，举全市之力精心筹办第16届亚运会和首届亚残运会，开闭幕式精彩绝伦，赛事运转顺畅有序，安保维稳万无一失，服务保障周到细致，筹办工作廉洁高效，实现“两个亚运、同样精彩”，赢得了国内外的高度评价和广泛赞誉。全市人民以实际行动诠释了“敢想会干为人民、和谐包容共分享”的广州亚运精神，进一步丰富了新时期广州城市精神内涵。“两个亚运”的成功举办，全面展示了广州改革开放和现代化建设的伟大成果，极大提升了广州的国际影响力、城市凝聚力和市民自豪感。

　　城市环境面貌实现“大变”。抓住筹办亚运的有利时机，以实现“天更蓝、水更清、路更畅、房更靓、城更美”为目标，大力实施城市环境面貌“大变工程”。落实“空气整治50条”和“新31条”措施，空气质量连续5年优于国家二级标准，2011年优良天数达到360天，摘掉戴了10 年的重酸雨区帽子。对全市127条河涌进行综合整治，建成白云湖、海珠湖，新建污水处理厂38座，铺设截污管网1234公里，城市生活污水处理率达88％，比2006年提高16.36个百分点，珠江广州段水质从劣五类提高到四类标准。建成西江引水工程，中心城区自来水出厂水质提前两年达到国家新标准。实施交通改善措施30条，新开通公交线路263条，市民出行条件逐步改善。对1500个社区进行人居环境综合整治。完成17个“城中村”改造方案，猎德村、黄埔古村等“城中村”改造已见成效，10万村民人居环境得到改善。实施《花园城市行动纲领》，推进青山绿地工程和城市绿化升级改造，新增和改造绿地面积246平方公里，全市森林覆盖率41.66％，建成区绿化覆盖率40.15％，人均公园绿地面积15.01平方米，建成1862公里绿道网。先后获国家园林城市、国家森林城市、国家环境保护模范城市、国家卫生城市等荣誉称号。

　　全国文明城市成功创建。深入推进社会主义核心价值体系建设，广泛开展现代公民教育，大力实施“迎亚运、讲文明、树新风、促和谐”全民行动，不断加强志愿服务工作，形成涵盖城乡家庭、社区乡村、企业单位、城区街镇的多层次文明创建网络，市民文明素质和城市文明程度进一步提升。2008年荣获首批“全国未成年人思想道德建设工作先进城市”。历经13年矢志不渝的努力，2011年以优异成绩荣获“全国文明城市”称号。

　　五年来，社会事业全面发展，民生福祉明显改善。

　　民生水平大幅提高。制定实施“惠民66条”及17条补充意见，城乡居民收入稳步提高，预计2011年城市居民人均可支配收入和农村居民人均纯收入分别达到34300元和14700元，比2006年提高14449元和6912元，五年年均增长11.6％和13.6％，农村居民收入增速连续4年高于城市居民。五年共帮扶36万名困难群众实现再就业。2011年全市城镇登记失业率控制在3.5%以内。社会救助水平不断提高，2011年城镇和农村低保平均标准达到467元和377元，分别比2006年提高45％和118％。五年共资助14.45万社会申办退休人员、8.15万困难企业退休人员和257万城镇居民参加基本医疗保险，新型农村合作医疗基本实现全覆盖，群众看病贵、看病难问题逐步缓解。完成1.64万户农村危破房改造。城镇独生子女父母计划生育奖励全面兑现，惠及15万群众。居民健康水平不断提高，城乡居民人均期望寿命从2006年的77.88岁提高到2010年的79.04岁。

　　民生实事全面兑现。认真办好十件民生实事，2011年十件实事全面完成：一是全面开展北部山区镇、贫困村对口帮扶工作，206个贫困村已有180个村年集体收入达到10万元以上，81％有劳动能力的贫困户人均年收入达到5000元以上。二是市财政安排4600万元，全面落实本市户籍贫困家庭学生免学费就读中等职业学校等事项。三是预计2011年五大险种新增参保人数178万人次，超过新增82万人次的目标要求；全市111万35岁以上农村居民全部纳入社会养老保险体系，提前一年完成省下达的任务。四是政府举办的基层医疗卫生机构全部实行国家基本药物制度；城镇居民基本医疗保险和新农合财政补助标准分别提高到200元和230元，新农合实际人均筹资额371元。五是城乡居民低保标准提高17％至25％；建立临时价格补贴与物价上涨联动机制，惠及近16万困难群众。7.7万名农村困难群众和重度残疾人获资助参加新农保；孤儿养育标准调高至每人每月1000元，并建立正常增长机制。六是筹集建设90281套保障性住房，完成目标任务的106％，提前一年解决在册的7.72万户低收入家庭住房困难问题。七是新建、改造城市道路81公里，新建人行过街设施27座。增设公交专用道超过70公里，在城乡结合部新开通100条公交线路，完成24个公交站场建设改造任务。八是80岁以上长者长寿保健金分段提高到每人每月100元、200元、300元，范围扩大至本市户籍70岁以上老年人和驻穗部队离退休干部，免费为80岁以上老年人安装“平安通”紧急呼援服务系统。九是完成5万盏农村路灯建设任务，全市30％的农村道路实现“亮化”。十是新建240处社区健身路径、188个社区篮球场和一批健身广场，新增绿道802公里，超额完成500公里的绿道建设任务。

　　社会事业全面进步。教育均衡发展水平不断提高，义务教育规范化学校覆盖率达84.51％，全面实现省教育强市、强区、强镇。中等职业教育和高等教育进一步发展，高技能人才队伍不断壮大，在世界技能大赛中获得好成绩。建成基本覆盖城乡的基层卫生服务机构。广州大剧院、辛亥革命纪念馆等一批公共文化设施落成，城市“10分钟文化圈”和农村“10里文化圈”基本建成。成功举办第九届中国艺术节、第八届少数民族运动会和中国音乐金钟奖，文艺创作精品纷呈。文化惠民工程扎实推进，广州被评为“最中国文化城市”之一。公益法律服务覆盖面不断扩大。群众体育活动蓬勃开展，五年来举办国际赛事57 项，我市运动员在国际赛事中勇夺2项奥运冠军和65项世界冠军，获第七届全国城市运动会金牌、奖牌、总分三项第一，广州足球首夺中超联赛冠军。圆满完成四川汶川抗震救灾和威州镇灾后重建任务，援藏援疆等对口支援工作扎实推进。投入帮扶资金16.86亿元，对口帮扶梅州、阳江、茂名三市，贫困户脱贫率达98.96％。我市行政首长连续两届当选世界城市和地方政府组织（UCLG）联合主席，国际友好城市和友好合作交流城市增至41个。国防动员、人民防空及优抚安置工作不断加强，连续6 次获“全国双拥模范城”称号。“扫黄打非”和打击走私成效显著。工会、共青团、妇联等人民团体联系群众的桥梁纽带作用进一步增强。连续五年被授予“广东省人口与计划生育先进单位”称号，成为国家人口计生综合改革示范市。荣获全国残疾人工作先进单位、全国民族工作先进单位和“爱心广州”等称号。民族宗教、司法行政、妇女儿童、侨务、贸促、对台、口岸、统计、档案、保密、参事、文史、修志、气象等各项工作取得新成绩。

　　五年来，宜居城乡建设取得新进展，城市承载力不断提升。

　　城市空间布局更加优化。深化实施“南拓、北优、东进、西联、中调”城市发展战略，编制完成《广州市城市总体规划纲要（2010－2020年）》，城市控制性详细规划覆盖十区，完成珠江新城-员村、琶洲地区、白云新城、白鹅潭地区、城市新中轴线地区、广州南站、广州（黄埔）临港商务区等重点功能区的规划编制并陆续推进建设，中新广州知识城建设已经启动，南沙新区新一轮开发建设拉开序幕。

　　基础设施建设加速推进。五年共投入2300亿元用于完善交通基础设施。建成南沙港区二期工程，预计2011年广州港货物吞吐量、集装箱吞吐量分别达到4.47亿吨和1440万标箱，分别居世界第6 和第7位。白云国际机场扩建工程加快推进，预计2011年机场旅客吞吐量突破4400万人次，位列世界前20名。五年共新开通120公里地铁、216公里高速公路、22.9公里快速公交（BRT）线路和1860公里市政道路。广州南站建成运营，快速轨道和高快速路网体系日趋完善。建成广州塔、花城广场等一批地标性建筑，中心城市功能进一步增强。

　　城市管理水平明显提升。完善以区为主体、多部门综合治理的城市管理机制。整治违法用地，依法拆除违法建设和户外违法广告招牌，实施“三线”下地，加强沙井盖与泥头车管理，有效遏制“六乱”现象，城市管理综合执法水平进一步提高。城镇生活垃圾分类处理稳步推进，无害化处理率超过90％。“菜篮子”工程建设成效明显，食品供应和价格调控有力。食品药品安全监管工作不断加强，遏制了重大食品安全事故。规范出租屋管理，流动人员服务管理水平进一步提升。社会治安综合治理成效显著，建立覆盖全市的治安视频监控系统，社会面防控体系不断完善，各类暴力犯罪案件持续下降，市民群众安全感进一步提升。加强安全生产监管，全市安全生产形势总体良好。应急管理体系进一步健全，成功化解2008年春运公共安全危机，妥善处置各类突发事件，社会大局保持和谐稳定。

　　新农村建设扎实推进。预计五年市本级财政累计投入涉农资金272.51亿元。完成113万亩农田水利标准化建设，整治“五小”水利工程285宗。中心镇建设稳步推进，全部行政村实现“五通”。开展村容村貌整治，初步实现农村生活垃圾统一收运和无害化处理。

　　五年来，重点领域改革和政府自身建设取得新突破，科学发展动力更加强劲。

　　各项改革不断深化。全面完成新一轮政府机构改革，加快行政审批制度改革，五年共清理行政审批项目近3000项，市级审批项目精简率达66.1%。实施简政强区（县级市）事权改革，推动64项事权下放。基本完成国有企业公司制改造，法人治理结构逐步完善。有效解决地方金融机构历史遗留问题，城市建设投融资体制不断完善。文化体制改革取得突破，市属国有经营性文化事业单位和8家文艺院团全部实行转企改制，组建一批大型国有文化企业集团。大力推动社会服务管理改革创新，街道“一队三中心”建设不断完善。社会组织快速发展，扶持培育力度不断加大。社区承担社会服务管理的职能进一步增强。增城市统筹城乡综合配套改革取得初步成效。

　　民主法制建设成效明显。认真贯彻市人大及其常委会决议和决定，依法定期向市人大及其常委会报告工作，自觉接受人大的法律监督、工作监督。认真接受市人大常委会组织的满意度测评和专题询问。定期向市政协通报工作，自觉接受市政协的民主监督。五年来，共组织实施17项代表议案决议，办理1792件人大代表建议、批评和意见，办理2500件政协提案，办复率达100％。开展“三级大接访”活动，完善市领导接访制度，畅通市长热线，开通“中国广州发布”官方微博，积极回应市民群众诉求。全面完成“五五”普法工作，连续3次荣获全国普法先进城市称号。五年共完成地方性法规草案19项，出台政府规章52项，修改和废止政府规章81项，法治环境更加完善。

　　政府自身建设进一步加强。深化政务公开，充分尊重民意，出台《广州市重大行政决策程序规定》和《广州市重大行政决策听证试行办法》，依法决策、科学决策、民主决策的能力不断提升。建成市政务服务中心，实施百项政府服务网上办理工程。加强公务员队伍建设，机关作风和行政效能进一步改善。在全国率先公布政府部门预算，2009年开始连续三年实现“三公”经费零增长，廉政建设取得新成绩。

　　回顾过去五年，我们深深体会到，做好政府工作，推动广州又好又快发展，必须坚持以科学发展观为统领，进一步解放思想，敢想会干，创造性地谋划广州的发展；必须坚持突出重点，攻坚克难，以抓落实论英雄，统筹推进各项工作；必须坚持以人为本，尊重民意，把民生幸福当作最高追求，着力增进民生福祉；必须坚持凝心聚力，团结奋进，着力培育城市精神，提高市民文明素养，形成建设幸福广州的强大合力；必须坚持深化改革开放，先行先试，增创体制新优势，为城市发展注入源源不断的新活力；必须坚持依法行政，廉洁高效，推动民主法制建设，彰显社会公平正义。

　　总结过去的工作，我们清醒地看到，我市经济社会发展仍然存在一些问题和薄弱环节。主要是：产业结构调整任务艰巨，自主创新能力和产业核心竞争力仍不够强；统筹城乡发展任务仍然十分繁重；城市管理科学化、精细化水平有待进一步提升，违法建设问题仍然较为突出；社会建设相对滞后，基本公共服务均等化还存在较大差距，社会服务管理还不适应社会结构的深刻变化；解决事关群众切身利益的住房、交通、教育、医疗、社会保障、环境保护等问题与市民的期盼还存在一定距离；政府自身建设和依法行政能力有待进一步加强。对此，我们必须高度重视，采取有力措施认真解决。

　　各位代表，五年的成绩来之不易，这是党中央、国务院和省委、省政府及市委正确领导的结果，是全市人民辛勤劳动、团结拼搏的结果，是社会各界大力支持的结果。在此，我谨代表广州市人民政府，向全市人民、驻穗解放军和武警部队官兵、各民主党派、人民团体以及社会各界人士表示崇高的敬意！向所有关心支持广州现代化建设的港澳同胞、台湾同胞、海外侨胞和国际友人表示衷心的感谢！

　　二、今后五年的奋斗目标和主要任务

　　今后五年，是我们全面建设国家中心城市，率先转型升级、建设幸福广州的关键时期。我们既面临重要战略机遇，也面临前所未有的困难与挑战。当前，世界经济复苏仍然存在许多不确定的因素。随着国家区域协调发展战略的深入实施，国内各地区科学发展势头迅猛，前有“标兵”、后有“追兵”，我们面临不进则退、慢进也是退的严峻形势。特别是亚运之后，广州要在新的起点上实现新发展，标准更高、难度更大。我们正处在转型发展的攻坚阶段，面临着公共服务供给与日益增长的民生需求不适应、城乡之间和区域之间发展不均衡、粗放式经济增长方式与城市核心竞争力提升不协调、单一的城市治理结构与多元利益格局不兼容等深层次矛盾。对此，我们必须保持清醒的头脑，进一步增强忧患意识，善于在大好形势中发现潜在危机，善于在前进道路上把握新的机遇，加强战略谋划，提高应对能力，奋力开创广州科学发展的新局面。

　　今后五年政府工作的总体要求是：高举中国特色社会主义伟大旗帜，以邓小平理论和“三个代表”重要思想为指导，深入贯彻落实科学发展观，全面实施珠三角《规划纲要》，按照市第十次党代会的决策部署，紧紧围绕全面建设国家中心城市的目标和率先转型升级、建设幸福广州的核心任务，以建设国际商贸中心和世界文化名城为战略重点，坚持低碳经济、智慧城市、幸福生活的城市发展理念，大力推进战略性基础设施、战略性主导产业、战略性发展平台实现重大突破，深化改革开放，科学规划、从容建设南沙新区，走新型城市化发展道路，全面提升城市核心竞争力、文化软实力和国际影响力，推动面向世界、服务全国的现代化国际大都市建设迈上新台阶。

　　今后五年经济社会发展的主要目标是：全面完成“十二五”规划，率先加快经济发展、城市发展、文化建设和社会建设转型升级，城市核心竞争力显著增强。综合经济实力持续增强，到2016年全市生产总值突破2万亿元，年均增长11％左右；转变经济发展方式取得重大进展，现代产业体系基本建立，具有全球辐射力的国际商贸中心、国际航运中心、先进制造业基地、区域金融中心和国家创新型城市建设取得新突破；改革开放进一步深化，城市活力增强，成为我国南方对外合作交流的核心门户；全省宜居城乡“首善之区”建设取得重大进展，以花城绿城水城为特点的生态城市基本建成；公共文化服务体系建设和文化产业竞争力位居全国前列，世界文化名城建设迈上新台阶；南沙新区开发建设取得重大进展，引领示范作用更加凸显；城乡一体化发展格局更趋完善，城乡居民收入差距进一步缩小，城市更加文明，市民更加富裕幸福，社会更加和谐稳定；民主法制更加健全，法治政府建设目标基本实现。

　　实现上述目标，必须深入贯彻落实市第十次党代会精神，紧紧围绕“12338”的决策部署，突出重点，把握关键，狠抓落实：“一个奋斗目标”，就是全面建设国家中心城市和率先转型升级、建设幸福广州，必须牢牢把握转型升级的战略机遇，强化高端要素集聚、科技创新和综合服务三大功能，增进民生福祉，提升城市核心竞争力。“两个战略重点”，就是要加快实现营商环境与国际接轨，打造具有较强全球辐射力的国际商贸中心；以弘扬岭南文化为核心，建设开放、包容、多元的世界文化名城。“三个城市发展理念”，就是要牢固树立低碳经济、智慧城市、幸福生活三位一体的理念，更加注重产业升级，更加注重科技创新，更加注重以人为本，实现包容发展，增强城市可持续发展能力。“三个重大突破”，就是集中力量、整合资源，努力在12类战略性基础设施、15项战略性主导产业、27个战略性发展平台建设上实现重大突破，大幅提升国家中心城市的综合承载力，整体提升产业核心竞争力。“八大工程”，就是要精心组织，强力推进产业提升、科技创新、城乡一体、生态环保、文化引领、人才集聚、民生幸福和党建创新八大工程，为走新型城市化发展道路、推动广州科学发展注入强大动力。

　　落实“12338”的决策部署，必须以世界眼光、战略思维、创新意识，把广州的发展置于世界先进城市的坐标体系中来谋划，以深化改革、扩大开放为根本动力，勇立潮头，大胆探索，创新发展路径，着力在“六个注重”上下工夫：一是注重提升产业核心竞争力。在做大经济规模的同时，突出提高经济发展质量。遵循低碳经济发展规律，实施产业升级工程，壮大优势产业，培育战略性新兴产业，提升传统产业，打造战略性发展平台，完善开放型创新体系，优化营商环境，加快现代产业体系建设，优化“广州服务”，壮大“广州制造”，提升“广州创造”和“广州质量”。二是注重城乡一体发展。完善统筹城乡发展体制机制，实施城乡一体工程，优化“一个都会区、两个新城区、三个副中心”的网络型城市空间布局，建设覆盖城乡的战略性基础设施，构建智慧型城市运行体系，提高资源利用效率，加强城市土地储备，建设人与自然和谐相处的生态城市，推动城市内涵发展，农村富裕发展，促进城乡共同繁荣。三是注重改善民生福祉。把民生幸福当作最高追求，实施民生幸福工程，大力推进基本公共服务均等化，办好民生实事，改革创新社区服务管理体制，形成城乡不同利益群体融合共处的局面，促进社会公平、正义、和谐，努力实现共同富裕，提升全体市民的幸福感。四是注重增强文化软实力。实施文化引领工程，深化文化体制改革，弘扬岭南文化，保护利用历史文化遗产，繁荣文化事业，做强文化产业，打造广州风格的城市名片，促进公共文化服务均等化、优质化，满足人民群众日益增长的精神文化生活需求。五是注重人才队伍建设。坚持人才为第一战略资源，实施人才集聚工程，优先发展教育，健全完善人才培养、引进、使用、激励的体制机制，统筹推进多层次人才队伍建设，激发城市发展活力。六是注重体制机制创新。深化制度改革，在重点领域、关键环节改革取得重大进展，进一步完善社会主义市场经济体制，建立政府、社会、市场良性互动的合作治理机制，提高市民对城市治理的参与度，优化现代城市治理结构，为实现科学发展提供体制机制保障。我们相信，经过五年的不懈努力，广州一定能够走出一条经济低碳、城市智慧、社会文明、生态优美、城乡一体、生活幸福的新型城市化发展道路，再创科学发展新辉煌。

　　三、2012年工作安排

　　2012年是新一届政府的开局之年。我们肩负着率先转型升级、建设幸福广州的新使命，承载着全市人民的新期待，必须切实按照市第十次党代会的决策部署，贯彻落实中央经济工作会议精神，坚持科学发展，更加坚定地走新型城市化发展道路，始终保持敢为人先的闯劲，始终保持攻坚克难的韧劲，始终保持为民造福的干劲，坚定信心，团结一致，抓住机遇，稳中求进，努力推动广州科学发展再上新台阶。

　　今年我市经济社会发展主要预期目标是：地区生产总值增长11％；全社会固定资产投资增长10％；社会消费品零售总额增长14.5％；外贸出口总值增长8.5％；万元生产总值能耗下降4.3％以上；二氧化硫、氮氧化物、化学需氧量和氨氮排放量分别下降2％、5％、3％和3％以上；城市居民人均可支配收入增长11％；农村居民人均纯收入增长12％；居民消费价格总水平涨幅控制在4％左右；城镇登记失业率控制在3.5％以内；人口自然增长率控制在7.2％。以内。重点做好以下九项工作：

　　（一）着力提高经济发展质量

　　保持经济平稳较快发展。全力推动总投资714亿元的101个重点项目建设，落实“新广州·新商机”推介会签约项目，确保项目按期开工、早日建成，促进投资稳定增长，增强经济发展后劲。开展“广货全国行”、“广货网上行”，提高广货市场占有率。进一步做旺旅游、美食、购物、会展等消费市场，培育文化娱乐、体育休闲等新型消费业态，促进消费持续增长。帮助企业疏通进出口渠道，促进对外贸易平稳发展。加强经济运行调节，营造良好发展环境。

　　加快推动产业转型升级。实施产业升级工程，制定配套政策，发挥每年40亿元财政扶持资金的引导作用，培育发展战略性主导产业，促进产业高端化、集群化和低碳化。壮大发展优势产业。重点发展商贸会展、金融保险、现代物流、文化旅游、商务与科技服务等5类现代服务业和汽车制造、石油化工、电子产品、重大装备等4类先进制造业。加快建设国际商品展贸城、珠影文化创意产业园、国际医药港等一批现代服务业载体项目。建设花都汽车制造基地100万辆乘用车和96万台汽车发动机、增城30万辆整车、广汽乘用车生产研发基地、南沙乘用车基地扩建项目，推进和谐型大功率机车检修基地、飞机维修基地等项目建设，促进新一代平板显示、宽带移动通信、数控机床等高技术产业集聚发展。培育发展战略性新兴产业。重点发展新一代信息技术、生物与健康、新材料产业与高端制造、时尚创意、新能源与节能环保、新能源汽车等6类新兴产业，建设国家集成电路设计产业化基地、光电科技产业基地、华南国际新材料产业等基地，培育新的增长点。改造提升传统产业。实施传统优势产业百强提升行动，巩固振兴广州“老字号”。推动中医药、钢琴、音响、皮革皮具、纺织、珠宝、食品等传统产业转型升级。擦亮“食在广州”金字招牌。优化改造商业批发和集贸市场，大力发展电子商务，推动“千年商都”向“现代商都”转型升级。优化产业空间布局。大力推进三大国家级开发区、六大现代服务业功能区、九大创新型产业发展区和九大功能性发展平台建设，为产业转型升级提供强大支撑载体。

　　促进外经贸转型发展和对外交流合作。优化出口结构，引导加工贸易企业加快由贴牌生产、委托设计向自有品牌营销等转型提升，推动贸易方式向货物贸易与服务贸易协调发展转变。优化市场结构，进一步巩固欧美日等发达经济体市场，拓展独联体、东盟、中东、非洲等新兴市场。优化利用外资结构，创新招商选资方式，继续办好“新广州·新商机”推介活动，吸引世界500强投资项目。推进跨境贸易人民币结算业务发展，支持企业“走出去”，实现国际化发展。深化穗港澳合作，扩大对台交流。发起设立“广州国际城市创新奖”，承办好世界大都市协会2012年董事年会，促进我市与世界各国城市间的友好交往。加快推进广佛同城化和广佛肇经济圈建设，促进广州与周边地区战略合作，统筹基础设施规划建设，实现产业错位协调发展。

　　倾力打造区域金融中心。推动广州国际金融城、广州金融创新服务区、广州碳排放权交易所、广州航运交易所等金融平台建设。支持金融机构做大做强，加快发展村镇银行、小额贷款公司和融资性担保机构。大力实施企业上市“双百工程”。积极发展股权投资市场，建设股权投资中心和财富管理中心。创新金融产品和服务，加快发展科技金融、汽车金融、贸易金融、航运物流金融、消费金融等业务，推进社区金融服务体系建设。加强与港澳台地区和国外金融合作。

　　助推企业做强做大。改善营商环境，维护公平竞争的市场规则。推进市属经营性国有资产统一监管，深化国有企业改革，完善董事会制度和企业考核激励机制，推进国有资产资本化、国有资本证券化。制定出台支持民营经济发展实施细则，支持总部企业、民营企业和中小微型创新企业发展。引导民营与外资企业参与战略性主导产业、战略性基础设施和战略性发展平台建设。选择100家企业给予重点扶持，争取一批企业进入全国企业500强和服务业企业500强，全面提升企业竞争力。

　　大力发展低碳经济。完善低碳经济政策法规体系，制定相应规划指引。建设一批低碳产业基地，打造低碳经济的龙头企业。继续推进“退二”企业搬迁和“双转移”工作，推动高耗能企业节能行动。围绕余热余压利用、机电节能改造、绿色照明等领域，推进低碳技术研发及应用。抓好省级循环经济试点，推广清洁能源，提高可再生能源比例，推进合同能源管理。实施建筑节能和技术创新示范工程，大力发展绿色建筑。倡导低碳生活方式，建设低碳社区，构建低碳交通体系，推行公共机构节能。

　　实施质量强市战略。牢固树立全面、全程、全员的质量意识，提高产品质量、工程质量、服务质量和环境质量，提升“广州质量”。推动企业采用国际标准和国外先进标准，资助企业参与国内外标准化活动，力争80家以上国内外标准化专业技术委员会秘书处落户广州，完成150项以上标准及技术规范制（修）订，叫响“广州标准”。大力发展工业设计。加快建设质量技术检验检测认证公共服务平台，新建2个国家质量检测中心。加大品牌培育力度，力争新增一批全国驰名商标和省级名牌产品，逐步培育一批世界名牌产品，增强广州国际竞争力。

　　（二）着力增进民生福祉

　　认真办好十件民生实事。一是稳定“菜篮子”价格。资助建设100家平价商店；对承担广州市场定向供应任务的种养基地、冷储基地和实行产销对接、农超对接的平价商店（专区）给予补贴；对低收入群体给予临时价格补贴。二是提高社会保障水平。将城镇职工月人均基本养老保险待遇提高8%至10%；建立重特大疾病医疗救助专项制度；城乡居民低保标准提高10%至14%；低收入困难家庭认定标准由当地低保标准的1.2倍提高到1.5倍；对“六类老人”政府购买居家养老服务标准按不同类别在原有基础上增加100元，增加为70周岁以上孤寡老人、60周岁以上重度残疾或仅与重度残疾子女居住的老人每人每月分别购买200元和300元的居家养老服务。三是提高公共卫生服务水平。市民基本公共卫生服务项目增加至11大项37小项；免费为1000例贫困白内障患者实施复明手术；免费为我市当年政策内怀孕生育的约10万户家庭提供出生缺陷干预服务。四是资助普惠型学前教育。对我市低收入困难家庭儿童、孤儿、残疾儿童以及其他优抚对象接受普惠型学前教育进行资助。五是加大住房保障力度。建立健全以公共租赁住房为主的保障性住房制度，筹集保障性住房4.5万套，新增解决1.5万户低收入家庭住房困难问题。六是促进就业。发放6万张职业技能培训券，引导本市劳动者通过参加职业培训提高就业能力；对吸纳本市就业困难人员再就业的用人单位，给予每人每月缴纳社会保险费用约350元补助，帮扶18万名登记失业人员实现再就业。七是继续做好北部山区镇村扶贫开发。年底所有被帮扶的贫困村年集体收入基本达到10万元以上，被帮扶的贫困户家庭人均年收入基本达到5000元以上；贫困镇的市政及公共服务设施水平有较大提升。八是改善出行条件。优化新增50条城乡公交线路，与地铁接驳的公交线路比例提升到55%以上；完善金沙洲、同德围道路基础设施；为主要地段人行过街天桥加建雨篷。九是改善社区便民服务。全市每个街道至少建成1个家庭综合服务中心；政府各部门实现网上办理的服务事项接入市民网页；为50个社区配套安装自助金融终端设备。十是丰富文化体育活动。新建50个500平方米以上社区文化广场；修建265条以上健身路径和126个篮球场，建设9项乡镇农民体育健身工程；新建绿道200公里，完善绿道的服务管理。

　　加快各项社会事业发展。落实积极就业政策，做好特殊困难群体的就业援助。加快建设市高技能人才公共实训鉴定基地，完善就读技工院校资助制度，加强对大学生、外来务工人员的就业指导，力争全年新增就业30万人。进一步完善劳动行政主管部门、工会、企业劳动关系三方协商制度，促进和谐劳资关系。大力推进国家、省教育体制改革试点。实施学前教育三年行动计划，积极构建学前教育公共服务体系，加大民办幼儿园扶持力度，发展公办幼儿园，确保2013年公办幼儿园比重达到30%以上。促进义务教育均衡发展，加强教师队伍建设，力争各区（县级市）通过义务教育规范化学校督导验收。健全特殊教育体系，完善促进民办教育发展和外来务工人员子女入学政策。推进教育国际合作，引进高端教育资源。严格落实校车安全等各项校园安全措施。深化医药卫生体制改革，进一步扩大基本药物制度实施范围，构建和谐医患关系。加强基层卫生服务体系建设，形成以全科医生为重点的基层医疗卫生队伍。积极应对人口老龄化挑战，完善城乡养老服务体系，加快市第二老人院和市级居家养老综合服务平台建设，鼓励社会力量参与福利机构建设运营。扶持发展慈善公益事业，广泛开展扶贫济困活动。健全完善残疾人服务体系。进一步发展志愿服务事业，打造“志愿者之城”。启动健康城市行动计划，倡导阳光健康的生活方式，大力推进全民健身，加大公共体育设施建设和开放力度，向市民提供更多的公共体育服务。提升竞技体育综合实力，大力发展体育产业，加强“无规定马属动物疫病区”的管理维护。推进羊城幸福家庭促进计划，提高出生人口素质。落实民族宗教政策，促进民族团结和宗教和睦。扎实开展“六五”普法工作。加强防灾减灾基础设施建设，提高“三防”工作水平。落实优抚安置政策，加强国防教育和“双拥”工作。做好海防、民防及打击走私工作。继续做好侨务、贸促、对台、气象、统计、档案、保密、参事、文史、修志等工作。

　　提高社区服务管理水平。以建设“幸福社区”为目标，推进社会服务管理重心下移，深化街道社区和镇村社会服务管理体制机制改革，完成街道“一队三中心”建设，开展特大镇、村社会服务管理改革创新试点，强化社区自治。深化社会组织登记管理体制改革，设立市、区两级社会组织培育发展专项资金，建设市、区社会组织培育基地，促进社会组织规范有序健康发展。鼓励支持社会组织参与政府购买服务，加强社会工作人才队伍建设。深化基层多元化服务改革创新，鼓励居民参与社区公共事务管理和公益事业，建立志愿者参与社区服务工作机制。继续推进社区信息服务“五个一”工程建设。积极发展家政服务业。加强外来务工人员服务管理，继续推行和完善流动人口“积分制入户”政策，推动就业指导、技能培训、子女教育、法律援助、社会保障等基本公共服务逐步向外来务工人员覆盖，积极吸纳外来务工人员参与社区、村镇社会服务管理。

　　（三）着力提升城乡建设管理水平

　　完善城乡规划和土地利用规划体系。完成《广州城市总体规划（2010－2020）》编制和上报工作，做好重点地区控制性详细规划和城市设计的编修。加强对重点地区的规划管理，科学编制轨道交通、供电、供水、环卫、人防、水系岸线等专项规划。完成以中新广州知识城和科学城为核心的东部山水新城总体规划。优化花都、增城、从化城市副中心规划，辐射带动镇村协调发展。完成市级重要景观街区、道路、节点的规划编制，深化历史文化名城保护，加强城乡规划和土地利用规划的衔接，更好地形成人口、产业、资源和环境相协调的城市空间格局。

　　节约集约利用土地资源。全面落实最严格的耕地保护和节约用地制度。推进全国城乡统筹土地管理制度创新试点，加快构建城乡一体的土地市场体系。创新土地储备机制。实施差别化和节约高效供地政策，优先保障重点区域、重点项目、重大产业、重要民生项目用地和农村集体征地留用地需求。推进集体建设用地流转，加强集体闲置土地、低效用地的盘活处置力度。用好城区关停工业企业的土地，提高土地节约集约利用水平。加强“三旧”改造的规划管理, 完善各类历史用地手续，大力推进“三旧”改造。

　　加快建设战略性基础设施。集中力量推进空港、海港、陆路交通、信息主枢纽、生态体系、市政设施、能源保障等12类战略性基础设施建设。重点推进白云国际机场扩建工程、广州港南沙港区三期、出海航道三期工程建设，建成南沙港粮食及通用码头。争取贵广、南广铁路广州段全线通车。加快建设南部、东部和北部综合交通枢纽，积极推动穗莞深城际、佛莞城际、广佛环城际等珠三角城际轨道交通线网建设，加快地铁六号线、七号线、九号线、广佛线广州段建设进度，尽快启动新一轮地铁线网建设。推进一批高快速道路建设，建成增从高速、广深沿江高速广州段二期。大力推进兆瓦级发电、110千伏及以上变电站、天然气高压管网等能源基础设施建设，增强发展保障能力。

　　提高城市管理精细化水平。按照标准化、常态化、网格化、智能化、人性化的要求，全面创新城市管理。实施“天网工程”，建立统一联动的数字化城市管理信息平台。加大打击假冒伪劣商品的力度，规范市场管理。健全食品药品监管体制，加大生产、流通、销售等各环节的联合执法力度，确保食品药品安全。强化企业安全生产主体责任，建立健全隐患排查治理体系。加强社会治安综合治理，严厉打击各类违法犯罪。深化应急管理体系建设。大力强化公交优先，推进城区交通改善措施，重点整治27个交通“黑点”，提高道路畅通水平。多措并举改善中心城区“停车难”问题。依法查处违法建设和违法广告招牌。持续整治“六乱”，进一步提升环卫保洁水平，做好迎接国家卫生城市复审工作。规范工地施工和泥头车运营管理。加大城市管理立法和综合执法力度，提高文明执法水平。

　　（四）着力推进国家创新型城市建设

　　建设智慧城市。以智慧型基础设施为根基，建设高性能宽带信息网络，加快推进“光纤到户”工程，筹建国家超级计算广州中心和国际云计算中心。以智慧型技术为主干，推动高端芯片研发及产业化，开展物联网、云计算等关键技术攻关。以智慧型产业为骨架，大力发展软件和信息服务业，建设国家电子商务示范城市，打造网络商都，建设中国软件名城。以智慧型应用为目标，初步实现公共安全、电网、地下管线管网、城市规划、土地利用总体规划、工程招投标等城市管理智能化。推进交通、医疗、社保等民生项目智能化。推进天河智慧城、中新广州知识城、南沙新区等智慧城市试点建设，在各区（县级市）开展智慧社区、智慧乡村、智慧楼宇等试点工作。

　　实施科技创新工程。加快推进战略性创新平台引进和建设，发展战略性新兴产业创新集群，培育科技小巨人企业，扶持创新型骨干企业发展，组织重大关键共性技术攻关，促进重大科技成果转化，引进创新创业团队和领军人才，培育珠江科技新星，开展重大国际科技合作，推进科技金融创新，集聚创新资源，全面提升城市创新能力。

　　打造国际创新人才高地。加强高层次、高技能人才队伍建设，拓展留交会功能，打造中国海外高层次人才交流第一品牌。积极开展国际人才交流合作，实施万名海外人才集聚工程、引进海外人才来穗创业红棉计划、创新创业领军人才百人计划和人才特区计划，进一步做好侨务招才引智工作。大力普及科学知识，培养青少年科技创新人才，提高全社会科学素养，营造有利于创新人才成长的发展环境。

　　完善开放型城市创新体系。加大科技研发投入，全市研发经费投入占地区生产总值比重达到2.26％以上。全力推动中新广州知识城建设，高标准建设基础设施，加强与新加坡的合作，开展知识经济创新发展模式试验，力争落成一批高端项目。推进中国科学院广州生物与健康研究院扩建工程、中国科学院广州技术研究院二期、中乌巴顿焊接研究院等重大科技创新平台建设，加快广州科学城的功能开发，高标准建设广州国际创新城、广州国际科技合作交流中心和广州国际科技孵化基地、广州国际生物岛。发展重点领域的产学研技术创新联盟，促进科技成果转移转化。支持企业设立技术中心，承担国家、省市重大科技攻关项目。建立科技创新风险保险机制，扩充和运用好市创业投资引导基金、科技担保等科技金融平台，带动社会资金投入创新型企业。加大专利申请资助和专利产业化引导投入，实施“保护知识产权市长奖”等激励政策，严厉打击侵权违法行为，营造知识产权保护的良好氛围。

　　（五）着力推进南沙新区开发建设

　　高标准做好新区规划。遵循“科学开发，从容建设”的核心理念，突出以人为本，宜居优先，处理好新城与老城、南沙与横琴和前海、南沙与周边地区、南沙与港澳的关系，推动完成《广州南沙新区发展规划》编制和报批工作，编制城市、产业、土地、交通、人口、生态等专项规划。优化产业布局，培育以先进生产性服务业为主导的产业高地，将南沙新区打造成为服务内地、连接港澳的商业服务中心、科技创新中心和教育培训基地，建设临港产业配套服务合作区、华南国际港务中心。大力发展海洋经济，使南沙新区成为国家实施海洋战略的重要基地。积极探索新型城市化发展道路，打造高品质岭南水乡之都，建成粤港澳全面合作综合示范区和优质生活圈，努力建设具有鲜明岭南特色的现代化滨海新城和面向未来的理想城市。

　　大胆先行先试。充分发挥南沙实施CEPA先行先试综合示范区的优势，推动与港澳的合作发展，率先形成与港澳接轨的营商环境。坚持政府引导与市场运作相结合，创新南沙新区的开发模式。大胆借鉴港澳等先进地区的管理机制，创新现代社会治理结构，开展社会服务管理创新试点。创新政策体系，推动南沙新区立法，为南沙新区长远发展提供政策法律保障。

　　扎实做好起步区建设。围绕60平方公里起步区建设，积极开展全球招商，大力引进高端优质项目。完善公共交通线网、高快速路、疏港铁路和海港、河港、航道等基础设施，打通连接珠三角周边城市和港澳地区的交通节点。完善生活配套设施，增强城市服务功能。开展低碳经济、智慧城市、生态城市试点，高标准高质量实施工程建设，使起步区成为南沙新区开发建设的示范。

　　（六）着力构筑岭南特色的生态城市

　　改善空气环境质量。加快广州市环境监测与预警中心建设，按照国家和省的要求，将PM2.5指标监测纳入空气质量评价体系。深化工业污染源治理，强化脱硫、脱硝、除尘设施的升级改造与运行管理，加大石化等重点行业污染防治力度。推广使用天然气等清洁能源，加快发展太阳能等新能源。加强机动车污染源头控制，全面实施国Ⅳ标准，严格执行黄标车限行措施。积极推广应用新能源汽车。继续深化餐饮业和工地扬尘污染控制，努力削减各类大气污染物排放。

　　深化水环境治理。建设大江大河防洪、防潮减灾体系，加强海洋环境监测和治理，推进江海堤防加固达标建设，实施水库除险加固工程。加快推进城乡供水应急备用水源、花都北江引水工程和中心城区北部水厂及配套管网建设。强化饮用水源保护，加强备用水源管理。加强重点污染源、污水处理厂在线监控。建立地表水环境长期动态监测系统，推进重点水库入库河流水质监测。加快河涌综合整治和生态修复，基本完成东濠涌二期、荔枝湾涌三期大地涌段工程。大力整治“水浸街”。完善截污管网，年内全市城市生活污水处理率达到89％以上。启动建设5个新湖、5个公共沙滩泳场，建设水上绿道。打造珠江、增江、流溪河、滨海岸线景观，凸显岭南水乡特色，建设生态“水城”。

　　加强废弃物处置和利用。大力开展环保宣传，提高市民环保意识。坚持减量化、资源化、无害化，推进生活垃圾分类处理，规范餐厨垃圾集中收集、运输和处理，实现建筑废弃物规范管理。加强再生资源回收利用，从源头推进垃圾减量。创新垃圾处理模式，鼓励社会力量参与垃圾处理。加快李坑、兴丰等垃圾填埋场、发电厂、综合处理厂和市废弃物安全处置中心建设，筹建番禺、南沙、花都、增城、从化生活垃圾焚烧发电厂和萝岗固体资源再生中心，力争城市生活垃圾无害化处理率达到92％，资源化利用率达到30％，生活垃圾人均日产量零增长，危险废弃物处置率保持100％。

　　提升城市森林绿化水平。完善森林、绿地、湿地、自然保护区和沿海防护林等自然生态系统建设，切实保护古树名木，构筑“一屏四片”生态屏障和“三纵五横”生态走廊，促进生物多样性。着力推进城市绿化美化和森林资源保护五大工程，建设好239万亩生态公益林。推进环城森林、组团绿化隔离带等生态景观林带连接贯通，确保森林覆盖率保持在41.66%以上。实施花园建设计划，每个区（县级市）兴建一个以上花园。实施城市主干道、人行天桥、建筑物天台、主要标志性建筑周边绿化美化。完善绿地系统和城市绿廊花廊建设，推进社区公园建设，推广种植乡土树种及彩叶和开花植物，形成森林围城、绿道穿城、绿意满城、四季花城的岭南园林景观，彰显“绿城”和“花城”风采。

　　开展生态城市建设试点。按照“一年试点先行，三年全面铺开，五年初具规模”的实施步骤，每个区（县级市）建设一个不同主题的生态示范区。增城、从化、花都重点建设生态田园城市示范区和生态旅游度假示范区，海珠重点开展万亩果园湿地保护区建设，番禺重点开展观光休闲农业示范区和水上绿道建设，三个国家级开发区重点建设生态工业示范园区，中心城区重点建设生态型社区，东部山水新城和南沙新区重点建设生态新城示范区。

　　（七）着力推动城乡一体化发展

　　发展都市型现代农业。大力推进从化万花园、南沙都市农业产业园、增城小楼人家、花都“花之都”等现代农业园区建设，重点建设水稻、蔬菜、水果、花卉、水产5个万亩示范基地和1个百万头生猪养殖基地。大力推广“公司＋基地＋合作组织＋农户”等新型农业产业化模式，大力支持农民专业合作社建设。深入实施新一轮“菜篮子”工程，建立市外供穗“菜篮子”生产基地，加大农超对接力度。加快发展种子种苗产业、观光休闲农业和生态农业，拓展农业功能。建立多层次、广覆盖的农业风险保障体系。完善农产品流通基础设施，加强农产品质量安全监管，维护农产品市场稳定。

　　实施美丽乡村行动计划。完善全市村镇规划体系，合理安排农田保护、村落分布、生态涵养等乡村空间布局，着力保护农村地区生态环境和文化资源。实施名镇名村工程，市重点推进3个名镇、4个名村建设。推进村庄整治，进一步改善农村人居环境，提高村镇保洁和绿化水平，增加农村休闲度假功能，打造优美宜居的都市乡村，引导和推动城乡生活双向互动。

　　加快农村基础设施升级改造。实施农村基础设施“五网”升级工程，全面完成新一轮1200公里村道建设任务。大力加强农田水利基础设施建设，稳步推进农村供水工程，建设北部山区43个贫困村生活污水处理设施。加快山区农村光纤入户，提高山区农村无线通讯服务质量。推广城乡生活垃圾统一收运处理，加快普及无害化粪池和卫生厕所。继续推进农村“光亮”工程二期建设，不断改善农村生产生活条件。

　　提高农村公共服务水平。开展新型农村社区建设，加快建设农村公共服务站，实施面向农村地区学校的“名校对口扶持工程”，提高农村义务教育水平。基本完成25所中心镇医院、镇卫生院和370个村卫生站标准化建设，构建农村30分钟医疗服务圈。完善农村社会救助体系。普及“农家书屋”，扩大农村“十里文化圈”覆盖面。加强农民就业技能培训，每年培训新型农民8000名。

　　完善城乡一体化长效机制。建立健全农村产权流转平台，基本完成农村集体土地登记发证，推进土地承包经营权、集体建设用地使用权、房屋所有权和集体林权“四权”合法流转。进一步完善农村征地补偿制度，完成兑现农村留用地历史欠账，完善农村宅基地管理，切实维护农民土地权益。整合城乡养老保险制度，统筹城镇居民基本医疗保险制度和新型农村合作医疗制度。建立市内扶贫开发长效机制，增强贫困镇村脱贫奔康“造血”功能。全面完成广州与梅州、阳江、茂名对口开展扶贫开发“双到”工作任务，做好援疆援藏等对口支援工作。

　　（八）着力提振广州城市精神

　　深化文明城市建设。文明无止境，创建不停步。进一步完善文明城市建设的长效机制，实现文明城市建设制度化、规范化、常态化，推动文明城市建设活动向镇村延伸、向深层次拓展。加强社会主义核心价值体系建设，培育新时期现代城市人文精神。发挥舆论在弘扬正气、扬善抑恶和道德伦理建设中的引导作用，加强社会公德、职业道德、家庭美德、个人品德和未成年人思想道德建设。构建社会诚信体系，倡导社会志愿服务和爱心互助，积极培育良好社会心态，在全社会推动形成知荣辱、讲道德、促和谐的文明风尚，整体提升市民素质和城市文明程度。

　　大力弘扬岭南文化。建立多层次的历史文化名城保护体系，深入挖掘、整合和利用岭南文化资源，重点打造海上丝绸之路、南越国历史文化、近现代革命史、华侨文化、宗教文化、广府文化和古城中心区文化品牌。按照“一山一江一城八个主题区域”的整体保护框架，加强对全市48片历史文化保护区的保护控制，重点加强长洲岛、黄埔古港古村、沙湾古镇、南海神庙、十三行、从化广裕祠等历史文化遗产保护和开发。做好传统历史建筑、特色商业街等保护利用。鼓励岭南文艺精品创作，大力扶持粤剧粤曲、广东音乐、岭南美术的发展。保护发展民俗文化，重视对“三雕一彩一绣”等非物质文化遗产的扶持、保护和传承。科学设计和规划建设特色鲜明的城市文化新地标，打造一批具有广州风格的城市名片，提升城市品质，彰显城市魅力，展现城市精神。

　　推动文化事业和文化产业大发展。深化文化体制改革，引导社会力量参与文化建设。加强对外文化交流与合作，扩大广州文化影响力和辐射力。完善基本公共文化服务体系，实施农村电影放映、文化消费补贴等文化惠民工程，丰富群众文化生活。建设广州博物馆新馆、农民工博物馆、南粤先贤馆、“团一大”纪念广场等重点文化设施，办好中国（广州）星海国际合唱锦标赛、羊城国际粤剧节、中国音乐金钟奖等重点文化节会活动。加强文化产业政策扶持，建设国家文化产业示范区，推进广州国际媒体港、广州文化创意产业中心等重大项目建设，重点发展文化创意、传媒出版、演艺娱乐等文化产业，建设文化创意之都。

　　（九）着力建设人民满意政府

　　努力建设法治政府。政府工作人员要带头学法、讲法、守法，增强法治意识，倡导尊重法律的社会风尚。认真执行市人大及其常委会各项决议和决定，切实做好代表议案决议的实施，代表建议、批评、意见的办理和人大常委会审议意见的研究处理工作。认真执行《中共广州市委政治协商规程（试行）》，自觉接受市政协的民主监督,主动听取社会各界的意见建议。落实《广州市重大行政决策程序规定》，扩大公众对政府决策的有效参与。加强政府立法工作，重点推动城市管理、社会建设和基本公共服务等领域的立法。坚持依法办事，严格行政执法。开展法治政府示范区和依法行政示范点创建活动，扩大法律援助和法律服务覆盖面，完善社会矛盾纠纷调解机制。

　　努力建设服务政府。强化政府在经济调节、市场监管、社会管理和公共服务方面的职能，促进政府职能转变。加快建设电子政务服务平台，实现90％的政府行政许可事项全流程网上办理。进一步深化行政审批制度改革，推进行政审批管理“零收费”制度试点，减少行政许可事项。完善责、权、利相统一的市、区（县级市）分工管理体制，推动管理重心下移。积极稳妥推进事业单位分类改革。加强机关作风建设，改善服务态度，提高服务水平。

　　努力建设阳光政府。坚持廉政亲民、勤政爱民、善政惠民。完善政务公开制度，促进行政权力运行过程和结果更加公开透明。健全新闻发布和发言人制度，充分利用政府微博等传播方式，拓宽政民互动渠道，推进网络问政。完善和落实部门预算公开制度。自觉接受市民群众监督和社会舆论监督，推动行政权力在阳光下健康运行，确保人民群众的知情权、表达权、参与权、监督权，促进社会公平正义。

　　努力建设廉洁政府。建立健全行政决策廉政风险防范机制。健全公共财政体制，优化财政支出结构。加强对政府投资项目招标投标、经营性土地使用权出让、政府采购等的监督检查。坚持厉行节约，勤俭办一切事情。严格控制“三公”经费支出使用，树立为民、务实、诚信、清廉的良好形象。

　　各位代表，开启新征程，实现新目标，关键在于抓落实。我们要牢固树立在抓落实上见分晓、比高低、论英雄的观念，加强队伍建设，转变工作作风，强化督查考核，全面提高政府执行力，切实履行政府职责。让我们更加紧密地团结在以胡锦涛同志为总书记的党中央周围，在市委的坚强领导下，紧紧依靠全市人民，同心同德，开拓进取，奋力拼搏，为率先转型升级、建设幸福广州而努力奋斗！
